# Long-term ozone exposures and cause-specific mortality in a US Medicare cohort

**DOI:** 10.1038/s41370-019-0135-4

**Published:** 2019-04-16

**Authors:** Fatemeh Kazemiparkouhi, Ki-Do Eum, Bingyu Wang, Justin Manjourides, Helen H. Suh

**Affiliations:** 1 Department of Civil and Environmental Engineering, Tufts University, Medford, MA USA; 2 College of Computer and Information Science, Northeastern University, Boston, MA USA; 3 Department of Health Sciences, Northeastern University, Boston, MA USA

**Keywords:** O_3_, Air pollution, Long-term exposure, Cause-specific mortality, Confounding

## Abstract

We examined the association of long-term, daily 1-h maximum O_3_ (ozone) exposures on cause-specific mortality for 22.2 million US Medicare beneficiaries between 2000–2008. We modeled the association between O_3_ and mortality using age-gender-race stratified log-linear regression models, adjusted for state of residence. We examined confounding by (1) adjusting for PM_2.5_ (particles with aerodynamic diameters <2.5 μm) and NO_2_ (nitrogen dioxide) exposures, temperature, and neighborhood-level characteristics and behaviors, and (2) decomposing O_3_ into its temporal and spatio-temporal components and comparing estimated risk ratios. We also examined sensitivity of our results to alternate exposure measures based on warm-season 8-h daily maximum and 24-h average exposures. We found increased risks from long-term O_3_ exposures to be strongest and most consistent for mortality from respiratory disease (1.030, 95% CI: 1.027, 1.034) (including COPD (chronic obstructive pulmonary disease)), CHF (congestive heart failure), and lung cancer (1.015, 95% CI: 1.010, 1.020), with no evidence of confounding by PM_2.5_, NO_2_, and temperature and with results similar across O_3_ exposure measures. While significant, associations between long-term O_3_ exposures and CVD (cardiovascular)-related mortality (1.005, 95% CI: 1.003, 1.007) were confounded by PM_2.5_ and varied with the exposure measure, with associations no longer significantly positive when warm-season 8-h maximum or 24-h average O_3_ was used to assess exposures. In this large study, we provide strong evidence that O_3_ exposure is associated with mortality from respiratory-related causes and for the first-time, lung cancer, but raise questions regarding O_3_-related impacts on CVD mortality. Our findings demonstrate the need to further identify potential confounders.

## Introduction

A small but growing number of studies have examined associations between long-term exposure to O_3_ and cause-specific mortality [1–4], with mixed results. In an analysis of the ACS (American Cancer Society) cohort, for example, Jerrett et al. [1] found significant associations between warm-season averages of 1-h daily maximum O_3_ exposures and increased CVD, IHD (ischemic heart disease), respiratory and cardiopulmonary mortality, but not all-cause mortality. However, with the exception of respiratory mortality, these associations disappeared after adjustment for PM_2.5_, suggesting confounding by PM_2.5_ of O_3_-mortality associations for cardiovascular-related causes [1].

Correspondingly, in a follow-up study of the ACS [2], associations of year round and warm-season averages of 8-h daily maximum O_3_ were significantly and positively associated with all-cause, circulatory, cardiovascular, and respiratory mortality. Unlike the earlier study, however, these associations, including that for CVD mortality, were not confounded by PM_2.5_. Findings from the CanCHEC study (Canadian Census Health and Environment Cohort) [3], on the other hand, showed significant and positive associations between warm-season 8-h daily maximum O_3_ and non-accidental, cardiometabolic disease, and cardiovascular mortality, but not respiratory, CBV (cerebrovascular disease), and lung cancer. Significant associations remained significant but were attenuated after adjustment for PM_2.5_ and NO_2_. In contrast, warm-season averages of 24-h averaged O_3_ exposure were not associated with all-cause, cardiovascular, CBV, non-malignant respiratory, and lung cancer mortality in the California Teacher’s Study [4]. While these studies suggest a relationship between average peak O_3_ exposures and mortality, their associations for specific causes of mortality are inconsistent, perhaps due to their use of different measures of long-term O_3_ exposure, which may not only affect O_3_’s impacts on health but also confounding of O_3_-mortality associations by PM_2.5_ and other pollutants.

Yet, few studies have examined how different O_3_ exposure measures impact associations with specific causes of death and how these impacts may affect control for potential confounders, such as PM_2.5_ and other air pollutants. Our ability to understand these impacts may be furthered by methods that assess unmeasured confounding in air-pollution-health effect models, such as that developed by Greven et al. [5]. We used this method to characterize unmeasured confounding in our recent studies of PM_2.5_ [6] and NO_2_-associated [7] mortality risks. We showed significant and positive associations between both PM_2.5_ and NO_2_ associations and several causes of death, even after adjustment for other air pollutants and neighborhood-level SES (socio-economic status) and behavioral factors; however, unmeasured confounding of these risks remained. No such method has yet been applied to O_3_ and cause-specific mortality.

To address this issue, we examined the association between long-term O_3_ exposures and all-cause and cause-specific mortality in a cohort of >22 million Medicare beneficiaries living across the conterminous US (United States) and assessed the consistency of these associations across different measures of exposure, control for confounders, and as modified by region of residence and urbanicity.

## Materials and methods

This study was approved by the Institutional Review Board of Tufts and Northeastern Universities.

### Air pollution data

We obtained hourly O_3_ concentrations from 1151 air quality monitors from the US EPA (Environmental Protection Agency) AQS (Air Quality System) for 2000 through 2008. These monitors, 80% of which were located in urban areas, were selected based on their having hourly measurements for 4 + calendar years, with each year having data for at least five warm-season months (April–September), with each month having at least 75% valid daily measurements. Daily measurements were determined to be valid if 75% of its hourly measurements were available. For each monitor and day, we calculated the daily maximum hourly O_3_ concentration as our main exposure measure and averaged daily values from April through September to obtain warm-season average, 1-h daily maximum O_3_ concentrations (hereafter referred to as the warm-season 1-h maximum O_3_) for each monitor and calendar year, as used in Jerrett et al. [1]. In addition, we calculated two secondary exposure measures, including the (1) warm-season average of daily 8-h maximum and (2) warm-season average of 24-h average.

To examine potential confounding, we also estimated (1) 1-year moving average PM_2.5_ concentrations using well validated GIS-based (Geographical Information System) spatio-temporal models that estimated daily PM_2.5_ exposures on a 6 km grid covering the conterminous US [8], (2) 1-year moving average NO_2_ concentrations using land use regression models developed by Bechle et al. [9] that estimated monthly NO_2_ exposure for census blocks, and (3) 3-day moving average temperature using spatio-temporal GAMs that estimated daily temperature on a 6 km grid [8]. PM_2.5_, NO_2_, and temperature values were linked to corresponding O_3_ exposures using the grid point or in the case of NO_2_, the census block closest to each of our study’s O_3_ monitors.

### Medicare beneficiaries

We compiled enrollment data from the Centers for Medicare and Medicaid Services for 52.9 million Medicare beneficiaries aged 65 to 120 between 2000–2008. We identified those beneficiaries that resided in ZIP (Zone Improvement Plan) codes with a geographic centroid within a 10 km of an eligible O_3_ monitor between 2000–2008. For each enrollee, we obtained information on date of birth, gender, race/ethnicity, ZIP code of residence, and survival. Using the ICD-9 (International Classification of Disease 9th version) codes from the National Death Index, we extracted mortality from non-accidental and accidental causes, as well as from all CVD (cardiovascular) and all respiratory-related causes, and specific subcategories, including IHD (ischemic heart disease), CBV (cerebrovascular) disease, CHF, COPD and pneumonia. Altogether, CVD and respiratory-related causes accounted for over 50% of all-cause mortality (Table 1). We computed the number of Medicare beneficiaries and cause-specific deaths for each 5-year age interval-gender-race (white/non-white) group, monitor, and study month. To avoid excessive zero counts, we collapsed ages 90 and above into one interval.

**Table 1:** Summary statistics of ozone monitors and concentrations, Medicare enrollees, and cause-specific deaths among Medicare enrollees

Characteristics	ICD−9 code	_25th_ Median _75th%_^a^	Total number	Total %
Monitoring stations			1151	
O_3_^b^(ppb)		_50_ 55 _60_		
Medicare enrollees		_3858_11,697 _28,311_	22,159,190	
All-cause deaths		_724_ 2,471 _6458_	5,766,091	
Non-accidental	A−R	_704_ 2,412 _6293_	5,637,693	97.8
Accidental	V−Y	_21_ 62 _155_	128,372	2.2
All cardiovascular	I00−I99	_279_ 956 _2537_	2,333,681	40.5
IHD	I20−I25	_136_ 461 _1226_	1,245,401	21.6
CBV	I60−I69	_55_ 183 _477_	410,187	7.1
CHF	I50	_21_ 73 _183_	158,649	2.8
All respiratory	J00−J99	_85_ 281 _725_	633,216	11.0
COPD	J40−J44	_73_ 155 _380_	328,957	5.7
Pneumonia	J12−J18	_20_ 67 _177_	174,932	3.0
All cancer	C−D	_156_ 534 _1437_	1,277,716	22.2
Lung cancer	C34	_46_ 155 _403_	350,357	6.1

Time period: 2000–2008, USA

*ICD-9* the 9th revision of the International Classification of Disease, *O*_*3*_ ozone, *IHD* ischemic heart disease, *CBV* cerebrovascular disease, *CHF* congestive heart failure, *COPD* chronic obstructive pulmonary disease

Medicare enrollees ages 65–120

^a^Values are medians among locations and months, with 25th and 75th percentile given in subscripts

^b^Warm-season average of daily 1-h maximum ozone concentrations

### Statistical analyses

We linked demographic and mortality data for our Medicare beneficiary cohort to O_3_ concentrations measured at the monitor closest to the centroid of the ZIP code of residence.

As our main analysis, we used log-linear regression models to examine the association between warm-season averages of 1-h maximum O_3_ exposure and cause-specific mortality, stratified on age, gender, and race (White/non-White) and adjusted for state of residence in both single and two-pollutant models controlling for 1-year moving average PM_2.5_ (Supplementary Appendix 1, SI (Supplementary Information)). All results are expressed as the RR (risk ratio) of dying in a given month per 10 ppb (part per billion) increase in O_3_ exposure and associated 95% CI (confidence interval).

We evaluated the sensitivity of our findings to the exposure measure, replacing warm-season 1-h maximum O_3_ concentrations with (1) warm-season average of daily 8-h maximum or (2) warm-season average of 24-h average O_3_ concentrations in our health models in single and two-pollutant models controlling for PM_2.5_. We also examined whether associations differed by region of residence (Northeast, Midwest, South, West) as classified using US Census Bureau classifications (Fig. S1, SI), and by urbanicity (urban vs. non-urban) using USGS (United States Geological Survey) Land Cover Trends classifications by stratifying our data into corresponding groups.

To further assess potential confounding of an association between O_3_ and mortality, we fit models that additionally adjusted for NO_2_ and 3-day moving average temperature. We also fit multi-variable models for beneficiaries living near the 836 of the 1151 monitors for which we had demographic covariates from the 2000 U.S. Census [10]. We assessed the association of O_3_ and mortality adjusting for PM_2.5_ with and without controlling for the proportion of the population ≥25 years old with at least a high school degree, median income, and proportion of the population living in urban area within the census tract corresponding to the ZIP code centroid. The spatial distribution of the monitors with Census data was similar to that for all monitors, although with fewer monitors in the Midwest (18% vs. 24% overall) and more monitors in the West (31% vs. 26% overall) (Table S1, SI).

We also examined potential confounding by behavioral covariates measured in Selected Metropolitan/Micropolitan Area Risk Trends of the BRFSS (Behavioral Risk Factor Surveillance System) [11] for the subset of beneficiaries living near monitors (586 of 1151 monitors) located in a county with BRFSS data. For these beneficiaries, we assessed the association of O_3_ and mortality, adjusting for PM_2.5_, and with and without adjusting for potential confounding by monthly county-level prevalence of current smokers, diabetics, heavy drinkers (i.e., >two drinks per day), asthma, average median income, and body mass index. Note that the spatial distribution of the monitors with BRFSS data was similar to that for all monitors, although with fewer monitors in the Midwest (16% vs. 24% overall) and more monitors in the West (31% vs. 26% overall) (Table S1, SI). The spatial distribution of the monitors with Census and BRFSS data was similar by region.

To examine the extent to which our findings remain affected by confounding, we followed the method suggested by Greven et al. [5] and decomposed O_3_ into two orthogonal, component measures, “temporal” and “spatio-temporal” O_3_, and estimated their associations with cause-specific mortality in base, PM_2.5_-adjusted, and BRFSS-adjusted models (Supplementary Appendix 1, SI). “Temporal” O_3_, which represents the national temporal trends in monthly O_3_ concentrations, is O_3_ centered by the average concentrations for all monitors and across the study period; “spatio-temporal” O_3_ is a measure of the monitor-specific temporal trends in monthly O_3_ concentrations compared to the national “temporal” trends. In the absence of residual confounding by long-term time trends of mortality, estimates of the “temporal” and “spatio-temporal” O_3_ should be similar.

## Results

Our study population included 22.2 million Medicare enrollees residing in 260 metropolitan areas across the US (Table 1), comprising 42% of all Medicare enrollees. During the study period, 5.8 million deaths were reported; 97.8% from non-accidental causes. CVD accounted for 40.5% of all mortality, followed by cancer (22.2%) and respiratory mortality (11%). More than 50% of all CVD mortality was caused by IHD. Fifty-two percent of respiratory deaths were attributed to COPD and 27.6% to pneumonia, while 27.4% of cancer deaths were attributed to lung cancer. The warm-season average of 1-h maximum O_3_ concentration across all monitors and years was 56 ppb (9 ppb) and exhibited no consistent temporal trend over the study period (Fig. S2, SI). Warm average concentrations varied by region, with concentrations highest in the West (Fig. S2, SI). Warm-season averages of 1-h maximum O_3_ were strongly correlated with corresponding averages of 8-h maximum (*r* = 0.98) and 24-h average (*r* = 0.74) levels. The correlation coefficient between the warm-season average of 1-h maximum O_3_ and 1-year average PM_2.5_, 1-year average NO_2_, and 3-day average temperature equaled 0.24, 0.26, and 0.04, respectively.

In single pollutant models we found a 10 ppb increase in warm-season 1-h maximum O_3_ to be significantly associated with an increased risk of dying from all (1.013; 95% CI: 1.012, 1.014) and non-accidental causes (1.014; 95% CI: 1.013, 1.015), but not from accidental causes (0.998; 95% CI: 0.991, 1.006) (Table 2). RRs were the highest for respiratory-related causes (1.036; 95% CI: 1.032, 1.039)., with O_3_ exposure linked to 1.065 times the risks of COPD (95% CI: 1.060, 1.069) and 1.024 times that of pneumonia (95% CI: 1.018, 1.030) deaths per 10 ppb increase in O_3_ exposures. Additionally, O_3_ exposures were associated with increased risk of CVD-related mortality (1.027; 95% CI: 1.025, 1.028). For specific causes of CVD mortality, an increase in warm-season 1-h maximum O_3_ exposure was significantly associated with 1.052 times the risk of CHF mortality (95% CI: 1.045, 1.060), 1.043 times the risks of IHD (95% CI: 1.041, 1.045) and 1.012 times that of CBV mortality (95% CI: 1.008, 1.016). One-hour maximum O_3_ exposure was also associated with increased risk of lung cancer mortality (1.016; 95% CI: 1.011, 1.020), but not mortality from all cancers (1.000; 95% CI: 0.998, 1.003).

**Table 2:** Mortality RRs (95% CI) associated with a 10 ppb increase in O_3_: single pollutant and PM_2.5_-adjusted models

Cause of death	Single pollutant model^a^	PM_2.5_-adjusted model^b^
All-cause	1.013 (1.012–1.014)	1.004 (1.003–1.006)
Accidental	0.998 (0.991–1.006)	1.010 (1.002–1.017)
All cardiovascular	1.027 (1.025–1.028)	1.005 (1.003–1.007)
IHD	1.043 (1.041–1.045)	1.008 (1.006–1.011)
CBV	1.012 (1.008–1.016)	0.993 (0.989–0.997)
CHF	1.052 (1.045–1.060)	1.063 (1.055–1.071)
All respiratory	1.036 (1.032–1.039)	1.030 (1.027–1.034)
COPD	1.065 (1.060–1.069)	1.072 (1.067–1.077)
Pneumonia	1.024 (1.018–1.030)	0.990 (0.984–0.996)
All cancer	1.000 (0.998–1.003)	0.995 (0.993–0.998)
Lung cancer	1.016 (1.011–1.020)	1.015 (1.010–1.020)

Time period: 2000–2008, USA

*RR* risk ratio, *CI* confidence interval, *PM*_*2.5*_ particles with aerodynamic diameters <2.5 μm, *IHD* ischemic heart disease, *CBV* cerebrovascular disease, *CHF* congestive heart failure, *COPD* chronic obstructive pulmonary disease

Risk ratios are age, gender and race stratified, and adjusted for state of residence

^a^Warm-season average of daily 1-h maximum ozone concentrations

^b^Models adjusted for 1-year moving average PM_2.5_ exposures

In models adjusting for PM_2.5_, O_3_-associated mortality risks remained significant and positive for all causes of death, except for CBV and pneumonia (Table 2). For several causes of death, PM_2.5_-adjusted associations were attenuated, with RRs decreasing slightly to 1.030 (95% CI: 1.027, 1.034) for respiratory mortality and more substantially to 1.005 (95% CI: 1.003, 1.007) for CVD mortality per 10 ppb increase in long-term O_3_. In contrast, for COPD and CHF mortality, RRs increased after adjustment for PM_2.5_, resulting in a 1.072 (95% CI: 1.067, 1.077) and 1.063 (95% CI: 1.055, 1.071) times increase in risk per 10 ppb increase in O_3_, respectively.

In models adjusting for NO_2_, O_3_-associated mortality risks remained unchanged for respiratory and lung cancer, increased slightly for CHF and COPD, and were slightly attenuated for other causes of death (Table S2, SI). Associations were generally robust to adjustment for temperature (Table S3, SI). As shown on Table S4 (SI), RRs of the PM_2.5_-adjusted association between warm-season 1-h maximum O_3_ and mortality were essentially unchanged after adjustment for potential confounding by ecologic covariates from the 2000 U.S. Census or county-level behavioral covariates from BRFSS.

When we examined the potential for unmeasured confounding, we found statistically significant, positive RRs for temporal O_3_ and negative RRs for spatio-temporal O_3_ in base models (Table 3). In two-pollutant models adjusting for PM_2.5_ (Table 3) and in multi-variable models adjusted for both PM_2.5_ and behavioral covariates (Table S5, SI), the magnitude of “temporal” RRs decreased for all causes, especially in the multi-variate model, but the “spatio-temporal” RRs remained unchanged.

**Table 3:** Mortality RRs (95% CI) associated with a 10 ppb increase in temporal and spatio-temporal O_3_: non-adjusted vs. PM_2.5_-adjusted model

Cause of death	Base model^a^		PM_2.5_-adjusted model^b^	
	Temporal	Spatio-temporal	Temporal	Spatio-temporal
All-cause	1.184 (1.177–1.190)	0.990 (0.988–0.993)	1.176 (1.170–1.182)	0.990 (0.988–0.993)
Accidental	0.979 (0.945–1.014)	1.004 (0.987–1.022)	0.982 (0.947–1.017)	1.004 (0.987–1.021)
All cardiovascular	1.430 (1.419–1.442)	0.980 (0.976–0.984)	1.408 (1.397–1.420)	0.980 (0.977–0.984)
IHD	1.570 (1.552–1.588)	0.978 (0.972–0.983)	1.536 (1.519–1.554)	0.979 (0.973–0.984)
CBV	1.518 (1.488–1.548)	0.977 (0.968–0.987)	1.489 (1.460–1.520)	0.978 (0.969–0.987)
CHF	1.137 (1.101–1.173)	0.991 (0.976–1.007)	1.138 (1.102–1.175)	0.991 (0.976–1.007)
All respiratory	1.178 (1.159–1.197)	0.985 (0.977–0.992)	1.170 (1.151–1.189)	0.985 (0.977–0.992)
COPD	1.059 (1.035–1.082)	0.991 (0.980–1.001)	1.057 (1.033–1.080)	0.990 (0.980–1.001)
Pneumonia	1.569 (1.521–1.617)	0.972 (0.958–0.986)	1.540 (1.493–1.589)	0.972 (0.959–0.986)
All cancer	1.131 (1.118–1.144)	0.990 (0.985–0.995)	1.126 (1.113–1.139)	0.990 (0.985–0.996)
Lung cancer	1.117 (1.093–1.141)	0.992 (0.982–1.003)	1.114 (1.090–1.138)	0.992 (0.982–1.003)

Time period: 2000–2008, USA

*RR* risk ratio, *CI* confidence interval, *PM*_*2.5*_ particles with aerodynamic diameters <2.5 μm, *IHD* ischemic heart disease, *CBV* cerebrovascular disease, *CHF* congestive heart failure, *COPD* chronic obstructive pulmonary disease

Risk ratios are age, gender and race stratified, and adjusted for state of residence

^a^Warm-season average of daily 1-h maximum ozone concentrations

^b^Models adjusted for 1-year moving average PM_2.5_ exposures

Risks of death were higher for beneficiaries living in non-urban as compared to urban areas for all-cause and CVD mortality, but were similar for respiratory and COPD mortality (Table S6, SI). For all causes of death, O_3_-associated mortality risks were highest in the Northeast and West as compared to the South and Midwest (Table S7, SI). Region-specific RRs were generally lower in PM_2.5_-adjusted as compared to non-adjusted models for all regions except the Northeast, for which RRs were generally comparable.

We examined the robustness of our results to the O_3_ exposure measure (Table 4). In PM_2.5_-adjusted models, RRs based on the warm-season average of 8-h maximum O_3_ were similar for mortality from all causes, CHF, all respiratory, COPD, and lung cancer, but were attenuated and no longer significant for CVD and IHD mortality [note that associations between warm-season 8-h maximum O_3_ and both CVD and IHD mortality were significant and positive in single pollutant models, suggesting confounding of 8-h maximum—but not 1-h maximum associations—by PM_2.5_ (Table S8, SI)]. Findings for warm-season averages of 24-h O_3_ mirrored those for 8-h maximum O_3_, although associations with all-cause mortality were no longer positive.

**Table 4:** Mortality RRs (95% CI) associated with a 10 ppb increase in different O_3_ exposure measures

Cause of death	1-h Max^a^ O_3_	8-h Max^a^ O_3_	24-h Average^a^ O_3_
All-cause	1.004 (1.003–1.006)	1.002 (1.001–1.003)	0.990 (0.988–0.991)
Accidental	1.010 (1.002–1.017)	1.018 (1.009–1.027)	1.025 (1.014–1.036)
All cardiovascular	1.005 (1.003–1.007)	0.997 (0.995–0.999)	0.973 (0.970–0.975)
Ischemic heart disease	1.008 (1.006–1.011)	0.996 (0.993–0.999)	0.964 (0.960–0.967)
Cerebrovascular disease	0.993 (0.989–0.997)	0.987 (0.982–0.991)	0.968 (0.962–0.974)
Congestive heart failure	1.063 (1.055–1.071)	1.072 (1.063–1.080)	1.066 (1.056–1.077)
All respiratory	1.030 (1.027–1.034)	1.033 (1.030–1.037)	1.021 (1.016–1.026)
COPD	1.072 (1.067–1.077)	1.084 (1.079–1.089)	1.084 (1.077–1.091)
Pneumonia	0.990 (0.984–0.996)	0.978 (0.972–0.985)	0.935 (0.927–0.944)
All cancer	0.995 (0.993–0.998)	0.993 (0.990–0.995)	0.983 (0.980–0.986)
Lung cancer	1.015 (1.010–1.020)	1.016 (1.011–1.021)	1.007 (1.000–1.014)

Time period: 2000 – 2008, USA

*RR* risk ratio, *CI* confidence interval, *IHD* ischemic heart disease, *CBV* cerebrovascular disease, *CHF* congestive heart failure, *COPD* chronic obstructive pulmonary disease

Risk ratios are age, gender and race stratified, and adjusted for state of residence and 1-year moving average PM_2.5_ exposures

^a^Warm-season average of daily ozone concentrations

## Discussion

In our cohort of over 22 million Medicare beneficiaries, we found consistent associations between long-term O_3_ exposures and increased mortality. Specifically, we found a 0.4%, 3.0%, 0.5%, and 1.5% increased risk in all-cause, respiratory, cardiovascular and for the first-time, lung cancer mortality, respectively, per 10 ppb increase in warm-season average, 1-h maximum O_3_ exposure in PM_2.5_-adjusted models.

Notably, we found increased mortality risks from long-term O_3_ exposures to be the strongest and most consistent for respiratory-related diseases (including all respiratory and COPD), CHF, and lung cancer mortality, with no evidence of confounding by PM_2.5_, NO_2_, or temperature or of sensitivity to the exposure measure. O_3_-associated RRs for respiratory-related mortality, for example, were not attenuated in models controlling for PM_2.5_ or NO_2_ and further were comparable for beneficiaries living in urban and non-urban areas, for which the composition of PM_2.5_ differs substantially [12, 13]. In contrast, associations between long-term O_3_ exposures and CVD-related mortality were less consistent, confounded by PM_2.5_ as evidenced by their attenuation in two-pollutant models and their stronger effects in non-urban as compared to urban environments. Further, we found O_3_-associated risks to vary with the exposure measure, with associations no longer significantly positive when warm-season 8-h maximum or 24-h average O_3_ was used to assess exposures, suggesting that long-term peak rather than average exposures reduce life expectancy. Altogether, these findings provide strong evidence that long-term peak O_3_ exposure is associated with mortality from respiratory-related causes and for the first-time lung cancer, but raise questions regarding O_3_-related impacts on CVD mortality.

Our findings were robust to further adjustment by ecological and behavioral covariates for all causes of death. They, however, likely remain affected by some unmeasured confounding, as suggested by the unequal coefficients for temporal and spatio-temporal O_3_. Our finding of unmeasured confounding, even after adjustment for PM_2.5_ and behavioral covariates, is not surprising given our reliance on administrative mortality data, which precludes us from controlling for covariates describing individual-specific SES, behaviors, and health histories, as was done in the CanCHEC study [3]. Despite this, findings from our study are consistent with previous studies of ozone and mortality, many of which did adjust for numerous individual-specific covariates. Of these studies, only Jerrett et al. [1] assessed O_3_ exposures for 448,850 ACS CPS-II (Cancer Prevention Study II) study participants as warm-season averages of daily 1-h maximum ozone concentrations, finding significant, positive associations for cardiovascular (1.011, 95% CI: 1.003, 1.023), IHD (1.015, 95% CI: 1.003, 1.026), respiratory (1.029, 95% CI: 1.010, 1.048) and cardiopulmonary (1.014, 95% CI: 1.007, 1.022) mortality as in our study, but not for all-cause mortality (1.001, 95% CI: 1.996, 1.007), in contrast to our study. Further, associations in the CPS-II cohort remained significant after adjustment for PM_2.5_ for mortality from respiratory but not from cardiovascular-related causes, which like our study suggests confounding by PM_2.5_ of O_3_-mortality associations for cardiovascular-related causes [1].

In a follow-up to the CPS-II study [2], the impact of long-term O_3_ exposures on cause-specific mortality was re-examined using new estimates of ambient O_3_, PM_2.5_, and NO_2_ concentrations, a larger number of participants, and an extended follow-up period. The new O_3_ exposure estimates were based on individual-specific measures of yearly and summer-only 8-h maximum O_3_ averaged between 2002–2004 concentrations, thus capturing spatial but not temporal variability in O_3_ exposures. As in the original CPS-II and in our study, the follow-up study found summer 8-h daily maximum O_3_ exposures to be significantly and positively associated with all-cause (1.02, 95% CI: 1.02, 1.03), respiratory (1.10, 95% CI: 1.07, 1.12), and COPD (1.08, 95% CI: 1.05, 1.12) mortality and consistent with the original CPS-II but not our study, reported null associations with lung cancer mortality. Null associations with lung cancer in the CPS-II studies may be attributed to the relatively small number of lung cancer deaths in CPS-II, even after extended follow-up, with a total of 16,432 as compared to over 350,000 lung cancer deaths in our study. Notably the extended CPS-II study also reported new, significant increased risks of CVD (1.02, 95% CI: 1.01, 1.04) and CBV (1.03, 95% CI: 1.01, 1.06) mortality, associated with summer average 8-h maximum O_3_, even after adjustment for PM_2.5_. It is possible, however, that their observed increased risks for CVD and CBV reflect incomplete control for confounding by PM_2.5_, given (1) their time invariant exposure assessment method, with both O_3_ and PM_2.5_ exposures estimated as the average value over multiple years, with the number of years differing substantially for the two pollutants, and (2) evidence of confounding of associations between O_3_ and CVD mortality by PM_2.5_ in our study and in the original CPS-II.

For mortality from all causes, CVD, IHD, CBV, pneumonia, and all cancers, our non-positive findings for warm-season averages of 24-h O_3_ in PM_2.5_-adjusted models are consistent with those from Lipsett et al. [4] in the California Teachers study, which reported null associations between summer averaged 24-h O_3_ and all-cause, cardiovascular, non-malignant respiratory, and CBV mortality, and significant associations with increased mortality for IHD in single, but not 2-pollutant models that included PM_2.5_. While Lipsett et al. found null associations between 24-h O_3_ and lung cancer, we found these associations to be significant and positive. The insignificant associations for lung cancer and other causes of death reported by Lipsett et al. [4] have been attributed to its relatively small sample size of approximately 125,000 women. Our results suggest that its use of warm-season averages of 24-h O_3_ as the exposure measure may also contribute to the observed null findings.

More puzzling are results from Crouse et al. [3], who used data from the CanCHEC to examine the association between warm-season averages of 8-h maximum O_3_ and a number of causes of death. The authors reported significant increased O_3_-associated risks of all-cause mortality (1.031, 95% CI: 1.026, 1.036), and null association for CBV (0.981, 95% CI: 0.961, 1.001) as in our study, but null associations with lung cancer (1.006, 95% CI: 0.990, 1.023) mortality and significant positive associations with mortality from CVD (1.037, 95% CI: 1.028, 1.047), and diabetes (1.156, 95% CI: 1.121, 1.190), even after adjustment for PM_2.5_ and NO_2_. The O_3_-associated risks of increased CVD mortality—together with observed null associations between PM_2.5_ and CVD mortality—caused the authors to hypothesize that the multi-pollutant results were affected by collinearity among the multiple pollutants and/or overlapping spatial patterns in O_3_ and PM_2.5_.

Support for this hypothesis is provided by our findings of effect modification of the O_3_-mortality relationship by region for each cause of death. One possible explanation for this region-specific variability in O_3_-mortality risks may be corresponding regional variability in the correlation between O_3_ and PM_2.5_, which may result in differential confounding by PM_2.5_ of regional O_3_ effects on mortality. Other factors that may contribute to observed heterogeneity in O_3_-mortality associations may also include regional differences in (1) PM_2.5_ composition, which may differentially confound O_3_-mortality associations, (2) air conditioning prevalence, which may contribute to differential exposure error, and (3) unmeasured confounders that were not captured by our control for strata for age, race, gender, and our adjustment for state of residence, PM_2.5_, census, and behavioral variables. Note that certain causes of death, such as pneumonia or CBV, may be more sensitive to the region-specific effects of confounding, due to their small number of deaths.

Importantly, our positive and consistent associations linking long-term peak O_3_ exposures to cardio-respiratory mortality are biologically plausible. Numerous studies have demonstrated the negative permanent effects of long-term exposure to O_3_ on pulmonary function and increased lung inflammation [14–22], which may lead to tissue injury [14], airway remodeling [15, 16], and pulmonary fibrotic changes [17]. Further, exposed animals were shown to exhibit similar or greater morphological and other changes after episodic as compared to continuous O_3_ exposures [18], lending support for our findings showing that warm-season averages of 1-h daily maximum O_3_ were more relevant to mortality as compared to 8-h daily maximum and 24-h average O_3_ exposures.

Our study has several limitations. First, we did not have information on beneficiaries’ activity and mobility patterns, which may contribute to exposure misclassification. Further, we used ambient, nearest monitor O_3_ concentrations to assess exposures, which are imperfect proxies of personal O_3_ exposures. This exposure error has been shown to bias RR estimates towards the null [23], possibly suggesting that the magnitudes of our associations are underestimated. We, however, minimized other sources of exposure error by (1) linking O_3_ exposure to beneficiary information by year in order to account for residential moves and ZIP code boundary changes and (2) using exposure values that were geographically close to residences, capturing exposures that result from nearby O_3_ emission sources, and thus reducing the potential for exposure misclassification. Second, although we did not have data on personal-level characteristics, we adjusted for county-level behavioral variables, including those related to smoking and comorbidities, with results essentially unchanged, consistent with little, if any, confounding of O_3_-mortality associations by the examined covariates. While our findings showed that adjusting for PM_2.5_ and behavioral covariates reduces unmeasured confounding, note that differences in the coefficients for temporal and spatio-temporal O_3_ remained, suggesting unmeasured confounding of the O_3_-mortality associations. These findings demonstrate the need for additional studies to examine the impact of unmeasured confounding on estimated risks posed by long-term O_3_ on mortality. Lastly, our findings may not be generalizable to younger age groups or to beneficiaries living away from monitors.

These limitations are balanced by the substantial strengths of our study and long time period of study. With more than 22 million Medicare beneficiaries with near 5.8 million deaths over the 9 years of study period, our study is well powered to detect meaningful associations for mortality from specific causes, allowing us to provide valuable, new information on the relationship between O_3_ exposures and specific CVD, respiratory and cancer-related deaths in addition to all-cause mortality.

## Supplementary information

Supplementary Material 1

Supplementary Material 2

Supplementary Figure S1

Supplementary Figure S2

Supplementary Information

Supplementary Table S1

Supplementary Table S2

Supplementary Table S3

Supplementary Table S4

Supplementary Table S5

Supplementary Table S6

Supplementary Table S7

Supplementary Table S8

## Data Availability

All analyses were performed using SAS 9.4 Software (SAS Institute Inc., Cary, North Carolina). SAS code used to generate the results is provided as supplementary information (Supplementary Appendix 2, SI).
