# Supplementary material for: Long-term ozone exposures and cause-specific mortality in a US Medicare cohort
Source: J Expo Sci Environ Epidemiol. 2019 Apr 16;30(4):650–8. doi: 10.1038/s41370-019-0135-4 (PMC7197379; doi:10.1038/s41370-019-0135-4)
Supplement: Supplementary file 1 — Supplementary Material 1 [file 41370_2019_135_MOESM1_ESM.docx]

# Detailed Methodology

## **Detailed Regression Analysis**

$logE\left( Y_{st}^{c} \right)=\log\left( N_{st}^{c} \right)+\log\left( h^{c}\left( s \right) \right)+\beta O_{t}^{c}$ [1]

where $Y_{at}^{c}$ is the number of deaths at age-gender-race strata *s* in month *t* for monitor *c*, $N_{dt}^{c}$ is the number of Medicare enrollees of age-gender-race strata *s* with a ZIP code of residence matched to location *c* at the beginning of the month *t*, and *β* is the increase in the log-hazard of dying in a given month for a 10 ppb increase in average O_3_ concentration during the calendar year.

## **Decomposition of O3 Exposure Measure into “Temporal” and “Spatio-temporal” Components**

We applied the method developed by Greven et al. [10] to decompose the O_3_ exposure into their “global” and “local” components, where:

- *“Temporal”*: represents the national temporal trends in monthly concentrations ($\bar{O_{t}}$) centered by the overall concentrations for all monitors and across the study period ($\bar{O}$):

$TemporalO_{t}= \bar{O_{t}}-\bar{O}$ [2]

Given that this measure describes national temporal trend, the “global” measure directly reflects long-term time trends in exposure.

- *“Spatio-temporal”*: is equivalent to centered O_3_ ${(O}_{t}^{C}-\bar{O_{c}})$ minus its global component $\left( \bar{O_{t}}-\bar{O} \right)$:

$Spatiotemporal O_{t}^{C}= {(O}_{t}^{C}-\bar{O_{c}})-(\bar{O_{t}}-\bar{O})$ [3]

where $\bar{O_{t}}$ represents the concentration mean for month *t* across all locations, $\bar{O_{C}}$ is the mean concentration for monitor *C*, and $\bar{O}$ the mean concentration across all monitors and years.
