# Supplementary material for: Long-term ozone exposures and cause-specific mortality in a US Medicare cohort
Source: J Expo Sci Environ Epidemiol. 2019 Apr 16;30(4):650–8. doi: 10.1038/s41370-019-0135-4 (PMC7197379; doi:10.1038/s41370-019-0135-4)
Supplement: Supplementary file 2 — Supplementary Material 2 [file 41370_2019_135_MOESM2_ESM.docx]

# **SAS Code**

/* SAS-code for: "Long-term ozone exposures and cause-specific mortality in a US Medicare cohort" */

/* Author: Fatemeh Kazemi and Ki-Do Eum */

/* Purpose: Fit model (1) in supplement to one region using the Backfitting algorithm described in

Section 2.4 in Greven's paper */

/* Variables */

/* site_id - unique identifier of each monitor (site) */

/* region - region as defined in the paper: 1, 2, 3, 4 */

/* year - year */

/* month - month */

/* o3maxh - warm season avg of 1-hr max o3 for each calender year at the given site */

/* ID - age-sex-race strata */

/* Ycause - number of death at each strata for each cause in month t for each location */

/* N - number of Medicare enrollees of each starta in each location at the beginning of the month */

/* state - state of residence */

/* period: 2000-2008 */

/* bufferzone: 6 mile radia */

/* monitors have data for at least 4 years, 5 warm months, 75% days in month & 75% hours in day */

/* enrollees from 65 to 120 years */

/* sex: female and male; */

/* race: White and Non-white; */

/* ctrling for state as class varaible */

/***********************************/

/***********************************/

libname center "/scratch/.../base";

ods listing;

**%macro** cause (cuz);

**%macro** region(rg);

data work0;

set center.o3cms_asr2008;

if &rg ne **1234** then do;

if region=&rg;

end;

run;

/****************************************************************************/

/* 2. Back Fitting **********************************************************/

/****************************************************************************/

/* STEP A ********************************************/

data merged;

set WORK0;

para=**1**;

offseta1=log(N);

site_idn=site_id***1**;

run;

**%macro** backfit (i,i2);

ods output parameterestimates = outpara;

title "effect of o3maxh";

proc genmod data=merged;

class state;

model Y&cuz. = o3maxh state / dist= poisson link=log offset=offseta&i ;

run;

title;

ods output close;

data VARbeta;

set outpara;

if parameter = "o3maxh";

o3maxhaB&i=estimate;

o3maxhaSE&i=StdErr;

o3maxhaP&i=probChisq;

keep o3maxhaB&i o3maxhaSE&i o3maxhaP&i;

run;

data paraoutma&i;

set VARbeta;

para=**1**;

run;

PROC PRINT DATA=PARAOUTMA&i;run;

/* storing the results */

Data results;

set VARbeta ;

region=&rg;

o3maxh_B = o3maxhaB&i ;* per Unit o3maxh;

o3maxh_SE = o3maxhaSE&i;* per Unit o3maxh;

o3maxh_p = o3maxhaP&i;

I=&i;

keep I o3maxh_B o3maxh_SE o3maxh_p region;

run;

proc append base=o3maxhEffect_ID_cat data=results force; run;

/*STEP B **********************************/

data merged;

merge merged paraoutma&i;by para;

offsetb&i= offseta1 + (o3maxh * o3maxhaB&i) ;

run;

**%macro** site_idn (site_idn);

data subset;

set merged;

if site_idn=&site_idn then output;

keep Y&cuz. ID offsetb&i site_id site_idn;

run;

proc sql noprint;

select count(*) into :observations from subset;

%if &observations > **0** %then %do;

proc genmod data=subset;

class ID;

model Y&cuz. = ID / dist= poisson link=log offset=offsetb&i ;

output out= logHa pred=pred;

run;

data logHa;

set logHa;

site_idn=&site_idn;

logpred_adj=log(pred)-offsetb&i;

keep ID logpred_adj site_id site_idn;

run;

proc sort data=logHa;by ID;run;

data logHaOne;

set logHa;

by ID;

if first.ID;

run;

proc append base=logHaAll data=logHaOne force; run;

title;

data subset;

stop;

set subset;

run;

%end;

**%mend** site_idn;

%***site_idn***(**010030010**); run;

**.**

**.**

**.**

%***site_idn***(**560391011**); run;

proc sort data=merged ;by site_id ID;

run;

proc sort data=logHaAll ;by site_id ID;

run;

data merged;

merge merged logHaAll;by site_id ID;

offseta&i2= log(N) + logpred_adj;

run;

proc datasets;

delete logHaAll;

run;

quit;

**%mend** backfit;

%***backfit*** (**1** , **2**);**run**;

%***backfit*** (**2** , **3**);**run**;

%***backfit*** (**3** , **4**);**run**;

%***backfit*** (**4** , **5**);**run**;

%***backfit*** (**5** , **6**);**run**;

**proc** **datasets**;

delete merged;

**run**;

**data** center.o3maxh&cuz&rg;

set o3maxhEffect_ID_cat ;

**run**;

**proc** **datasets**;

delete o3maxhEffect_ID_cat;

**run**;

**%mend** region;

%***region***(**1234**);

%***region***(**1**);

%***region***(**2**);

%***region***(**3**);

%***region***(**4**);

**%mend** cause;

%***cause*** (all); %***cause*** (nacc); %***cause*** (acc);

%***cause*** (cvd); %***cause*** (ihd); %***cause*** (cbv); %***cause*** (chf);

%***cause*** (resp); %***cause*** (copd); %***cause*** (pneu);

%***cause*** (canc); %***cause*** (lungc);
