# Supplementary figures and images for: Long-term ozone exposures and cause-specific mortality in a US Medicare cohort
Source: J Expo Sci Environ Epidemiol. 2019 Apr 16;30(4):650–8. doi: 10.1038/s41370-019-0135-4 (PMC7197379; doi:10.1038/s41370-019-0135-4)

**Figure S1.** Boundaries of the four geographical regions used for the analysis, 2000-2008, US.

**
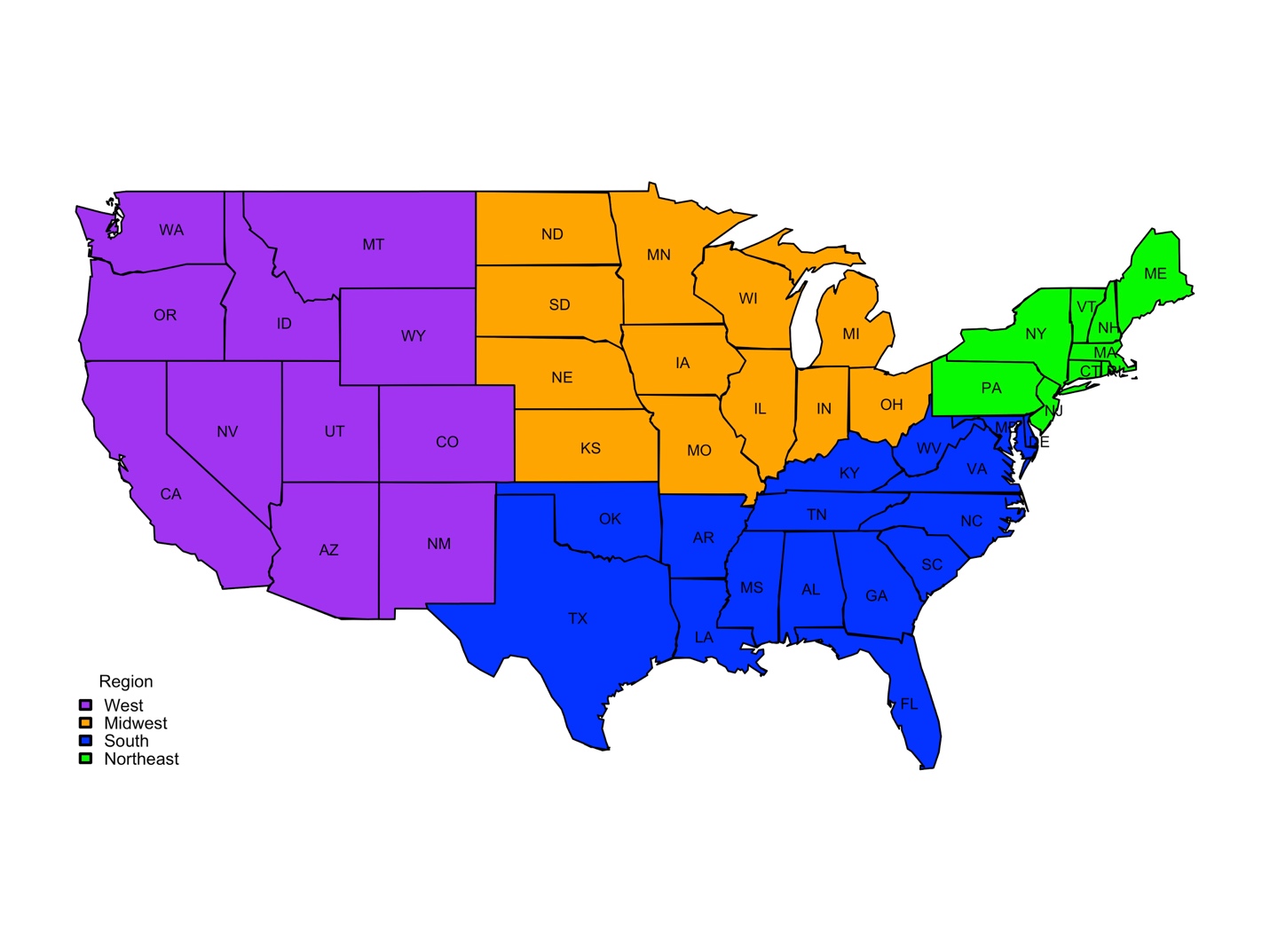
**

Supplement: Supplementary file 3 — Supplementary Figure S1 [file 41370_2019_135_MOESM3_ESM.docx]

**Figure S2**. Warm-season average O3 levels over the study period.


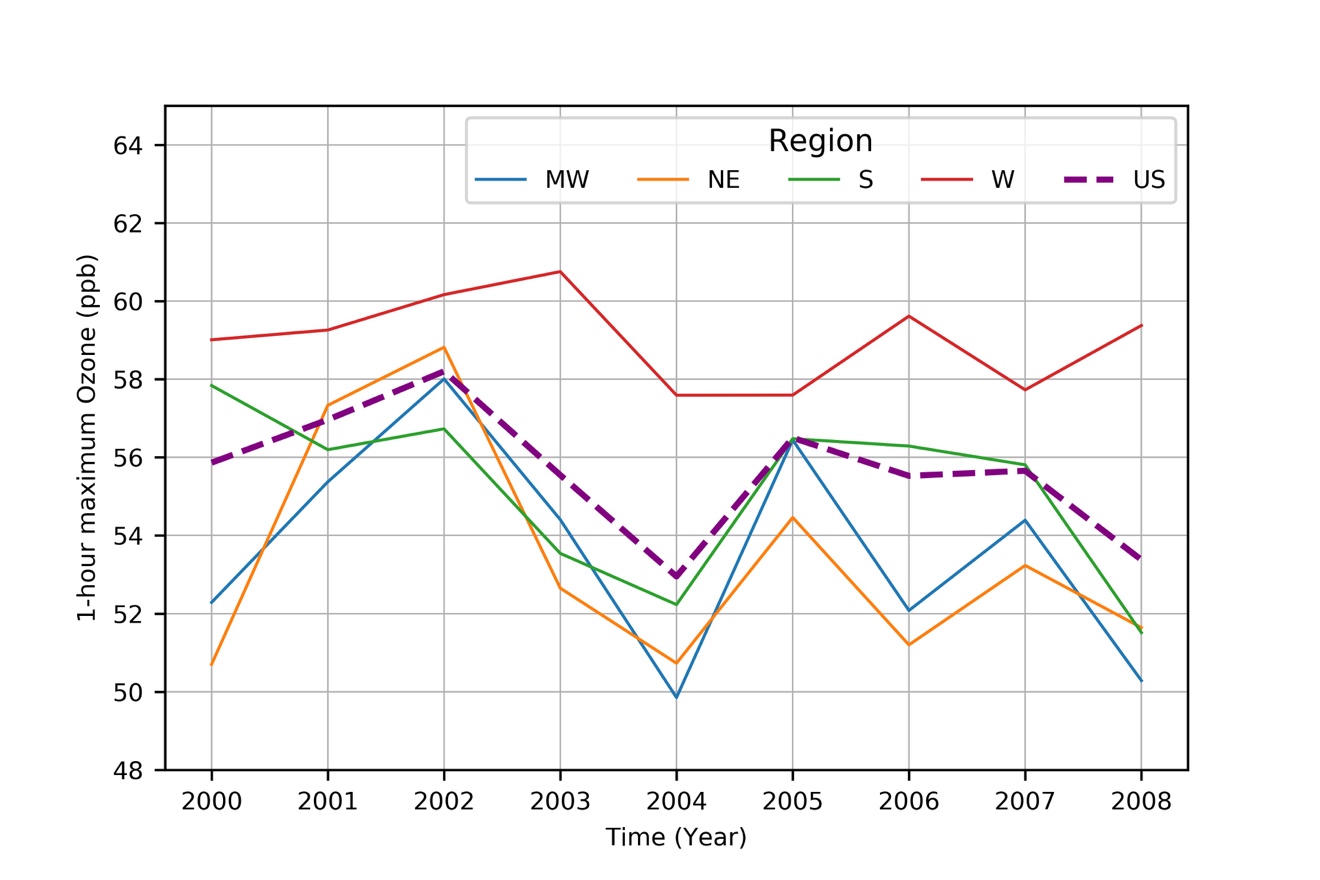

Supplement: Supplementary file 4 — Supplementary Figure S2 [file 41370_2019_135_MOESM4_ESM.docx]
