# Supplementary material for: Long-term ozone exposures and cause-specific mortality in a US Medicare cohort
Source: J Expo Sci Environ Epidemiol. 2019 Apr 16;30(4):650–8. doi: 10.1038/s41370-019-0135-4 (PMC7197379; doi:10.1038/s41370-019-0135-4)
Supplement: Supplementary file 5 — Supplementary Information [file 41370_2019_135_MOESM5_ESM.docx]

Long-term ozone exposures and cause-specific mortality in a US Medicare cohort

# Supplementary Information

| **Appendix 1** | Detailed methodology |
| --- | --- |
| **Table S1** | Distribution of O_3_ monitors across the US, by data availability. |
| **Table S2** | Mortality RRs (95% CI) associated with a 10 ppb increase in O_3_: single pollutant and NO_2_-adjusted models for monitors with NO_2_ data. |
| **Table S3** | Mortality RRs (95% CI) associated with a 10 ppb increase in O_3_: single pollutant and temperature-adjusted models. |
| **Table S4** | Mortality RRs (95% CI) associated with a 10 ppb increase in O_3_: PM_2.5_-adjusted versus full adjusted models for monitors with Census and BRFSS data respectively. |
| **Table S5** | PM_2.5_-adjusted mortality RRs (95% CI) associated with a 10 ppb increase in temporal and spatio-temporal O_3_, for a subset of monitors with BRFSS data |
| **Table S6** | Mortality RRs (95% CI) associated with a 10 ppb increase in O_3_: urban vs non-urban |
| **Table S7** | Mortality RRs (95% CI) associated with a 10 ppb increase in O_3_: by region |
| **Table S8** | Mortality RRs (95% CI) associated with a 10 ppb increase in different O_3_ exposure |
| **Figure S1** | Boundaries of the four geographical regions used for the analysis, 2000-2008, US. |
| **Figure S2** | Warm-season average O_3_ levels over the study period. |
| **Appendix 2** | SAS code used to generate the results. |
