# Supplementary material for: Long-term ozone exposures and cause-specific mortality in a US Medicare cohort
Source: J Expo Sci Environ Epidemiol. 2019 Apr 16;30(4):650–8. doi: 10.1038/s41370-019-0135-4 (PMC7197379; doi:10.1038/s41370-019-0135-4)
Supplement: Supplementary file 6 — Supplementary Table S1 [file 41370_2019_135_MOESM6_ESM.docx]

**Table S1.** Distribution of O_3_ monitors across the US, by data availability.

| **Region** | **Number of Monitors (%)** | | |
| --- | --- | --- | --- |
|  | **O_3_ Monitors** | **O_3_ Monitors**  **with Census** | **O_3_ Monitors with BRFSS** |
| **US** | 1151 (100) | 836 (100) | 586 (100) |
| West | 300 (26.1) | 261 (31.2) | 184 (31.4) |
| Midwest | 276 (24.0) | 150 (18) | 91 (15.5) |
| South | 417 (36.2) | 315 (37.7) | 220 (37.6) |
| Northeast | 158 (13.7) | 110 (13.1) | 91 (15.5) |
