# Supplementary material for: Long-term ozone exposures and cause-specific mortality in a US Medicare cohort
Source: J Expo Sci Environ Epidemiol. 2019 Apr 16;30(4):650–8. doi: 10.1038/s41370-019-0135-4 (PMC7197379; doi:10.1038/s41370-019-0135-4)
Supplement: Supplementary file 7 — Supplementary Table S2 [file 41370_2019_135_MOESM7_ESM.docx]

**Table S2.** Mortality RRs^1^ (95% CI) associated with a 10 ppb increase in O_3_^2^: single pollutant and NO_2_-adjusted models for monitors with NO_2_ data.

| **Cause of Death** | **Single Pollutant Model** | **NO_2_-adjusted Model^3^** |
| --- | --- | --- |
| **All-Cause** | 1.017 (1.016-1.018) | 1.014 (1.013-1.015) |
| Accidental | 0.999 (0.991-1.007) | 1.006 (0.998-1.014) |
| **All Cardiovascular** | 1.035 (1.034-1.037) | 1.028 (1.026-1.029) |
| IHD | 1.055 (1.053-1.058) | 1.043 (1.040-1.045) |
| CBV | 1.021 (1.017-1.025) | 1.016 (1.012-1.021) |
| CHF | 1.058 (1.051-1.066) | 1.066 (1.059-1.074) |
| **All Respiratory** | 1.039 (1.036-1.043) | 1.039 (1.036-1.043) |
| COPD | 1.067 (1.062-1.072) | 1.074 (1.069-1.079) |
| Pneumonia | 1.031 (1.025-1.037) | 1.016 (1.010-1.023) |
| **All Cancer** | 1.003 (1.000-1.005) | 1.001 (0.999-1.003) |
| Lung Cancer | 1.016 (1.011-1.021) | 1.017 (1.012-1.022) |

Abbreviations: RR = risk ratio; CI = confidence interval; PM_2.5_ = particles with aerodynamic diameters <2.5 μm; IHD = Ischemic heart disease; CBV = Cerebrovascular disease; CHF = Congestive heart failure; COPD = chronic obstructive pulmonary disease.

Time period: 2001 – 2008, US.

^1^ Risk ratios are age, gender and race stratified and adjusted for state of residence.

^2^ Warm season average of daily one-hour maximum ozone concentrations.

^3^ Models adjusted for 1-year moving average NO_2_ exposures.
