# Supplementary material for: Long-term ozone exposures and cause-specific mortality in a US Medicare cohort
Source: J Expo Sci Environ Epidemiol. 2019 Apr 16;30(4):650–8. doi: 10.1038/s41370-019-0135-4 (PMC7197379; doi:10.1038/s41370-019-0135-4)
Supplement: Supplementary file 8 — Supplementary Table S3 [file 41370_2019_135_MOESM8_ESM.docx]

**Table S3.** Mortality RRs^1^ (95% CI) associated with a 10 ppb increase in O_3_^2^: single pollutant and temperature-adjusted models.

| **Cause of Death** | **Single Pollutant Model** | **Temperature-adjusted Model^3^** |
| --- | --- | --- |
| **All-Cause** | 1.013 (1.012-1.014) | 1.016 (1.015-1.017) |
| Accidental | 0.998 (0.991-1.006) | 0.999 (0.992-1.006) |
| **All Cardiovascular** | 1.027 (1.025-1.028) | 1.030 (1.028-1.031) |
| IHD | 1.043 (1.041-1.045) | 1.046 (1.044-1.049) |
| CBV | 1.012 (1.008-1.016) | 1.015 (1.011-1.018) |
| CHF | 1.052 (1.045-1.060) | 1.055 (1.047-1.062) |
| **All Respiratory** | 1.036 (1.032-1.039) | 1.040 (1.037-1.044) |
| COPD | 1.065 (1.060-1.069) | 1.068 (1.064-1.073) |
| Pneumonia | 1.024 (1.018-1.030) | 1.031 (1.025-1.037) |
| **All Cancer** | 1.000 (0.998-1.003) | 1.001 (0.999-1.003) |
| Lung Cancer | 1.016 (1.011-1.020) | 1.016 (1.012-1.021) |

Abbreviations: RR = risk ratio; CI = confidence interval; PM_2.5_ = particles with aerodynamic diameters <2.5 μm; IHD = Ischemic heart disease; CBV = Cerebrovascular disease; CHF = Congestive heart failure; COPD = chronic obstructive pulmonary disease.

Time period: 2000 – 2008, US.

^1^ Risk ratios are age, gender and race stratified and adjusted for state of residence.

^2^ Warm season average of daily one-hour maximum ozone concentrations.

^3^ Models adjusted for 3-days moving average ambient temperature exposures.
