# Supplementary material for: Long-term ozone exposures and cause-specific mortality in a US Medicare cohort
Source: J Expo Sci Environ Epidemiol. 2019 Apr 16;30(4):650–8. doi: 10.1038/s41370-019-0135-4 (PMC7197379; doi:10.1038/s41370-019-0135-4)
Supplement: Supplementary file 9 — Supplementary Table S4 [file 41370_2019_135_MOESM9_ESM.docx]

**Table S4.** Mortality RRs^1^ (95% CI) associated with a 10 ppb increase in O_3_^2^: PM_2.5_-adjusted versus full adjusted models for monitors with Census and BRFSS^3^ data respectively.

| **Cause of Death** | **Census** | | **BRFSS** | |
| --- | --- | --- | --- | --- |
|  | **PM_2.5_-adjusted** | **Fully Adjusted^4^** | **PM_2.5_-adjusted** | **Fully Adjusted^5^** |
| **All-Cause** | 1.004 (1.003-1.005) | 1.006 (1.005-1.007) | 1.009 (1.007-1.011) | 1.007 (1.005-1.009) |
| Accidental | 1.011 (1.002-1.019) | 1.010 (1.002-1.019) | 0.995 (0.981-1.008) | 0.996 (0.982-1.010) |
| **All Cardiovascular** | 1.003 (1.002-1.005) | 1.004 (1.002-1.006) | 1.013 (1.009-1.016) | 1.010 (1.007-1.013) |
| IHD | 1.008 (1.005-1.010) | 1.010 (1.007-1.012) | 1.013 (1.009-1.017) | 1.010 (1.006-1.014) |
| CBV | 0.991 (0.987-0.996) | 0.989 (0.984-0.993) | 1.019 (1.012-1.027) | 1.016 (1.009-1.024) |
| CHF | 1.062 (1.054-1.070) | 1.061 (1.053-1.070) | 1.039 (1.026-1.052) | 1.036 (1.023-1.049) |
| **All Respiratory** | 1.031 (1.027-1.035) | 1.028 (1.024-1.032) | 1.035 (1.029-1.042) | 1.034 (1.028-1.040) |
| COPD | 1.075 (1.070-1.081) | 1.072 (1.066-1.077) | 1.058 (1.050-1.067) | 1.056 (1.048-1.065) |
| Pneumonia | 0.983 (0.977-0.990) | 0.981 (0.975-0.988) | 1.020 (1.009-1.032) | 1.019 (1.008-1.030) |
| **All Cancer** | 0.996 (0.994-0.999) | 0.998 (0.995-1.000) | 1.004 (0.999-1.008) | 1.002 (0.998-1.007) |
| Lung Cancer | 1.018 (1.013-1.023) | 1.019 (1.014-1.024) | 1.017 (1.009-1.026) | 1.017 (1.008-1.025) |

Abbreviations: RR = risk ratio; CI = confidence interval; IHD= Ischemic heart disease; CBV= Cerebrovascular disease; CHF = Congestive heart failure; COPD = chronic obstructive pulmonary disease.

Time period: 2000 – 2008, US.

^1^ Risk ratios are age, gender and race stratified and adjusted for state of residence.

^2^ Warm season average of daily one-hour maximum ozone concentrations.

^3^ BRFSS data first became available in 2002.

^4^ Models adjusted for PM_2.5_ and Census variables: proportion of population who are ≥25 years old with high school degree and above within the census tract, median income of the census tract associated with the beneficiary’s residence, proportion of population living in urban area within the census tract.

^5^ Models adjusted for PM_2.5_ and BRFSS variables: county-level smoking, diabetes, body mass index, alcohol consumption (>two drinks/day), asthma, and median income.
