# Supplementary material for: Long-term ozone exposures and cause-specific mortality in a US Medicare cohort
Source: J Expo Sci Environ Epidemiol. 2019 Apr 16;30(4):650–8. doi: 10.1038/s41370-019-0135-4 (PMC7197379; doi:10.1038/s41370-019-0135-4)
Supplement: Supplementary file 10 — Supplementary Table S5 [file 41370_2019_135_MOESM10_ESM.docx]

**Table S5.** Full adjusted mortality RRs^1^ (95% CI) associated with a 10 ppb increase in temporal and spatio-temporal O_3_^2^, for a subset of monitors with BRFSS data.

| **Cause of Death** | **Base Model** | | **Fully Adjusted Model^3^** | |
| --- | --- | --- | --- | --- |
|  | **Temporal** | **Spatio-temporal** | **Temporal** | **Spatio-temporal** |
| **All-Cause** | 1.117 (1.108-1.126) | 1.004 (1.000-1.008) | 1.105 (1.096-1.113) | 1.004 (1.000-1.008) |
| Accidental | 0.977 (0.928-1.028) | 1.009 (0.981-1.038) | 0.976 (0.927-1.028) | 1.008 (0.981-1.037) |
| **All Cardiovascular** | 1.226 (1.210-1.241) | 1.005 (0.998-1.012) | 1.193 (1.178-1.208) | 1.006 (0.999-1.013) |
| IHD | 1.285 (1.263-1.307) | 1.005 (0.996-1.014) | 1.241 (1.220-1.263) | 1.006 (0.997-1.015) |
| CBV | 1.204 (1.168-1.242) | 1.025 (1.009-1.042) | 1.165 (1.130-1.202) | 1.025 (1.009-1.042) |
| CHF | 1.172 (1.118-1.229) | 0.974 (0.950-0.999) | 1.156 (1.102-1.212) | 0.975 (0.950-0.999) |
| **All Respiratory** | 1.114 (1.088-1.141) | 1.006 (0.993-1.019) | 1.104 (1.078-1.131) | 1.006 (0.993-1.019) |
| COPD | 1.054 (1.020-1.090) | 1.007 (0.989-1.025) | 1.053 (1.018-1.089) | 1.007 (0.989-1.025) |
| Pneumonia | 1.325 (1.266-1.388) | 1.008 (0.984-1.033) | 1.287 (1.228-1.348) | 1.008 (0.984-1.033) |
| **All Cancer** | 1.085 (1.068-1.103) | 1.004 (0.995-1.013) | 1.078 (1.060-1.096) | 1.004 (0.995-1.013) |
| Lung Cancer | 1.101 (1.066-1.136) | 1.005 (0.988-1.022) | 1.094 (1.060-1.130) | 1.005 (0.988-1.022) |

Abbreviations: RR = risk ratio; CI = confidence interval; PM_2.5_ = particles with aerodynamic diameters <2.5 μm; IHD= Ischemic heart disease, CBV= Cerebrovascular disease, CHF = Congestive heart failure, COPD = chronic obstructive pulmonary disease.

Time period: 2002 – 2008, US.

^1^ Risk ratios are age, gender and race stratified and adjusted for state of residence.

^2^ Warm season average of daily one-hour maximum ozone concentrations.

^3^ Models adjusted for 1-year moving average PM_2.5_ exposures and BRFSS variables: county-level smoking, diabetes, body mass index, alcohol consumption (>two drinks/day), asthma, and median income
