# Supplementary material for: Long-term ozone exposures and cause-specific mortality in a US Medicare cohort
Source: J Expo Sci Environ Epidemiol. 2019 Apr 16;30(4):650–8. doi: 10.1038/s41370-019-0135-4 (PMC7197379; doi:10.1038/s41370-019-0135-4)
Supplement: Supplementary file 11 — Supplementary Table S6 [file 41370_2019_135_MOESM11_ESM.docx]

**Table S6.** Mortality RRs^1^ (95% CI) associated with a 10 ppb increase in O_3_^2^: urban vs non-urban

| **Cause of Death** | **Urban** | **Non-urban** |
| --- | --- | --- |
| **All-Cause** | 1.002 (1.001-1.003) | 1.016 (1.008-1.024) |
| Accidental | 1.009 (1.001-1.017) | 0.993 (0.946-1.042) |
| **All Cardiovascular** | 0.999 (0.998-1.001) | 1.036 (1.023-1.048) |
| IHD | 1.001 (0.999-1.003) | 1.054 (1.037-1.072) |
| CBV | 0.988 (0.984-0.992) | 1.042 (1.014-1.071) |
| CHF | 1.042 (1.014-1.071) | 1.062 (1.054-1.070) |
| **All Respiratory** | 1.039 (0.995-1.084) | 1.028 (1.025-1.031) |
| COPD | 1.028 (1.025-1.031) | 1.023 (1.000-1.045) |
| Pneumonia | 1.072 (1.067-1.077) | 1.013 (0.984-1.042) |
| **All Cancer** | 0.980 (0.974-0.986) | 1.075 (1.027-1.126) |
| Lung Cancer | 0.994 (0.992-0.996) | 1.006 (0.989-1.023) |

Abbreviations: RR = risk ratio; CI = confidence interval; IHD= Ischemic heart disease; CBV= Cerebrovascular disease; CHF = Congestive heart failure; COPD = chronic obstructive pulmonary disease.

Time period: 2000 – 2008, US

^1^ Risk ratios are age, gender and race stratified and adjusted for state of residence and 1-year moving average PM_2.5_ exposures.

^2^ Warm season average of daily one-hour maximum ozone concentrations.
