# Supplementary material for: Long-term ozone exposures and cause-specific mortality in a US Medicare cohort
Source: J Expo Sci Environ Epidemiol. 2019 Apr 16;30(4):650–8. doi: 10.1038/s41370-019-0135-4 (PMC7197379; doi:10.1038/s41370-019-0135-4)
Supplement: Supplementary file 12 — Supplementary Table S7 [file 41370_2019_135_MOESM12_ESM.docx]

**Table S7.** Mortality RRs^1^ (95% CI) associated with a 10 ppb increase in O_3_^2^: by region

| **Cause of Death and** | **Single Pollutant Model** | **PM_2.5_-adjusted Model^3^** |
| --- | --- | --- |
| **Region** |  |  |
| **All Cause** |  |  |
| West | 1.014 (1.013-1.016) | 1.008 (1.007-1.010) |
| Midwest | 1.008 (1.004-1.011) | 1.006 (1.002-1.010) |
| South | 1.013 (1.010-1.016) | 0.993 (0.991-0.996) |
| Northeast | 1.022 (1.018-1.025) | 1.024 (1.021-1.028) |
| **Accidental** |  |  |
| West | 0.987 (0.978-0.996) | 1.008 (1.007-1.010) |
| Midwest | 1.016 (0.991-1.042) | 1.006 (1.002-1.010) |
| South | 1.007 (0.990-1.025) | 0.993 (0.990-0.996) |
| Northeast | 1.003 (0.978-1.029) | 1.025 (1.021-1.028) |
| **All Cardiovascular** |  |  |
| West | 1.030 (1.028-1.032) | 1.008 (0.998-1.019) |
| Midwest | 1.012 (1.006-1.018) | 1.018 (0.992-1.044) |
| South | 1.026 (1.021-1.030) | 0.997 (0.979-1.015) |
| Northeast | 1.043 (1.037-1.049) | 1.005 (0.979-1.031) |
| **IHD** |  |  |
| West | 1.065 (1.062-1.068) | 1.009 (1.007-1.011) |
| Midwest | 0.988 (0.979-0.996) | 1.009 (1.003-1.015) |
| South | 1.019 (1.013-1.024) | 0.993 (0.988-0.997) |
| Northeast | 1.048 (1.040-1.055) | 1.047 (1.042-1.053) |
| **CBV** |  |  |
| West | 0.984 (0.980-0.989) | 1.026 (1.023-1.029) |
| Midwest | 1.048 (1.033-1.063) | 0.985 (0.977-0.993) |
| South | 1.056 (1.046-1.066) | 0.986 (0.980-0.993) |
| Northeast | 1.075 (1.060-1.090) | 1.054 (1.046-1.061) |
| **CHF** |  |  |
| West | 1.058 (1.049-1.067) | 0.969 (0.965-0.974) |
| Midwest | 1.052 (1.030-1.075) | 1.046 (1.031-1.061) |
| South | 1.033 (1.016-1.050) | 1.009 (0.999-1.020) |
| Northeast | 1.052 (1.030-1.074) | 1.079 (1.064-1.095) |
| **All Respiratory** |  |  |
| West | 1.035 (1.031-1.039) | 1.093 (1.082-1.103) |
| Midwest | 1.033 (1.021-1.045) | 1.052 (1.030-1.074) |
| South | 1.028 (1.020-1.037) | 1.015 (0.998-1.033) |
| Northeast | 1.040 (1.029-1.051) | 1.057 (1.035-1.079) |
| **COPD** |  |  |
| West | 1.077 (1.071-1.082) | 1.031 (1.027-1.035) |
| Midwest | 1.021 (1.005-1.038) | 1.033 (1.021-1.044) |
| South | 1.033 (1.021-1.044) | 1.015 (1.007-1.024) |
| Northeast | 1.074 (1.057-1.092) | 1.043 (1.031-1.054) |
| **Pneumonia** |  |  |
| West | 1.017 (1.010-1.024) | 1.085 (1.079-1.091) |
| Midwest | 1.077 (1.053-1.103) | 1.022 (1.006-1.038) |
| South | 1.044 (1.027-1.062) | 1.040 (1.027-1.052) |
| Northeast | 1.002 (0.982-1.021) | 1.080 (1.062-1.097) |
| **All Cancer** |  |  |
| West | 0.996 (0.993-0.998) | 0.977 (0.970-0.984) |
| Midwest | 0.993 (0.985-1.001) | 1.077 (1.052-1.102) |
| South | 1.018 (1.012-1.023) | 0.998 (0.980-1.016) |
| Northeast | 1.010 (1.003-1.017) | 1.010 (0.991-1.030) |
| **Lung Cancer** |  |  |
| West | 1.004 (0.999-1.010) | 0.996 (0.993-0.999) |
| Midwest | 1.009 (0.994-1.025) | 0.991 (0.983-0.999) |
| South | 1.036 (1.025-1.047) | 1.003 (0.997-1.009) |
| Northeast | 1.038 (1.023-1.053) | 1.012 (1.004-1.019) |

Abbreviations: RR = risk ratio; CI = confidence interval; PM_2.5_ = particles with aerodynamic diameters <2.5 μm; IHD= Ischemic heart disease; CBV= Cerebrovascular disease; CHF = Congestive heart failure; COPD = chronic obstructive pulmonary disease.

Time period: 2000 – 2008, US

^1^ Risk ratios are age, gender and race stratified and adjusted for state of residence

^2^ Warm season average of daily one-hour maximum ozone concentrations.

^3^ Models adjusted for 1-year moving average PM_2.5_ exposures.
