# Supplementary material for: Long-term ozone exposures and cause-specific mortality in a US Medicare cohort
Source: J Expo Sci Environ Epidemiol. 2019 Apr 16;30(4):650–8. doi: 10.1038/s41370-019-0135-4 (PMC7197379; doi:10.1038/s41370-019-0135-4)
Supplement: Supplementary file 13 — Supplementary Table S8 [file 41370_2019_135_MOESM13_ESM.docx]

**Table S8.** Mortality RRs^1^ (95% CI) associated with a 10 ppb increase in different O_3_^2^ exposure.

| **Cause of Death** | **1-hour Max O_3_** | **8-hour Max O_3_** | **24-hour Average O_3_** |
| --- | --- | --- | --- |
| **All-Cause** | 1.013 (1.012-1.014) | 1.008 (1.007-1.009) | 0.983 (0.981-0.984) |
| Accidental | 0.998 (0.991-1.006) | 1.011 (1.002-1.019) | 1.034 (1.023-1.045) |
| **All Cardiovascular** | 1.027 (1.025-1.028) | 1.011 (1.009-1.013) | 0.956 (0.954-0.959) |
| IHD | 1.043 (1.041-1.045) | 1.017 (1.014-1.019) | 0.934 (0.931-0.938) |
| CBV | 1.012 (1.008-1.016) | 1.000 (0.996-1.005) | 0.959 (0.953-0.964) |
| CHF | 1.052 (1.045-1.060) | 1.067 (1.058-1.075) | 1.074 (1.063-1.084) |
| **All Respiratory** | 1.036 (1.032-1.039) | 1.038 (1.034-1.042) | 1.016 (1.011-1.021) |
| COPD | 1.065 (1.060-1.069) | 1.081 (1.075-1.086) | 1.085 (1.078-1.092) |
| Pneumonia | 1.024 (1.018-1.030) | 1.000 (0.993-1.006) | 0.911 (0.903-0.920) |
| **All Cancer** | 1.000 (0.998-1.003) | 0.996 (0.993-0.999) | 0.980 (0.976-0.983) |
| Lung Cancer | 1.016 (1.011-1.020) | 1.017 (1.012-1.022) | 1.006 (0.999-1.013) |

Abbreviations: RR = risk ratio; CI = confidence interval; IHD= Ischemic heart disease; CBV= Cerebrovascular disease; CHF = Congestive heart failure; COPD = chronic obstructive pulmonary disease.

Time period: 2000 – 2008, US.

^1^ Risk ratios are age, gender and race stratified and adjusted for state of residence.

^2^ Warm season average of daily ozone concentrations.
